# Harnessing genetic potential to advance sustainability in beef systems

**DOI:** 10.1093/af/vfag031

**Published:** 2026-06-19

**Authors:** Matthew A Cleveland, Julie Ojango, Raphael Mrode, Mike Coffey, Troy Rowan

**Affiliations:** ABS Global, De Forest, WI; Animal Genetic Resource Utilization and Management, International Livestock Research Institute, Nairobi, Kenya; Animal Genetic Resource Utilization and Management, International Livestock Research Institute, Nairobi, Kenya; Animal and Veterinary Sciences, Scotland’s Rural College, Edinburgh, UK; Animal and Veterinary Sciences, Scotland’s Rural College, Edinburgh, UK; Department of Animal Science, University of Tennessee, Knoxville, TN, USA

**Keywords:** beef, genetics, phenotyping, selection, sustainability

## Abstract

**Graphical Abstract:**
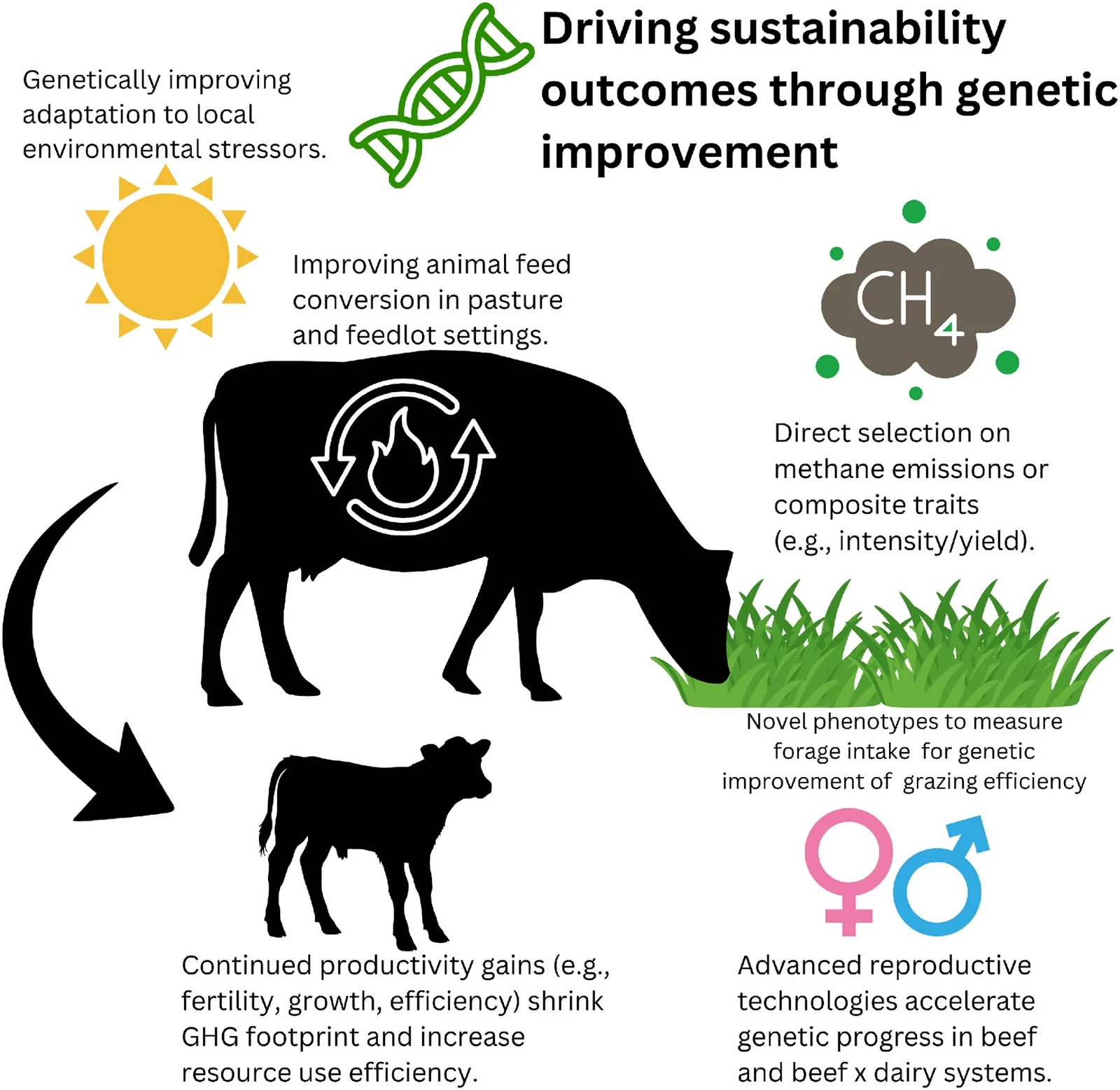

Implications

- Genetic selection offers global beef systems an opportunity to generate permanent, cumulative improvement in traits that impact sustainability.
- The collection of phenotypes is the major bottleneck for developing new selection tools for sustainability traits.
- Direct selection to reduce enteric methane emissions is possible, but it will require clearer economic signals before it is included in breeding objectives.
- The maintenance of local adaptation is an essential pillar of sustainability, especially in production systems in the Global South.

## Introduction

Global beef systems have undergone enormous changes in recent decades, steadily increasing in outputs while holding inventory relatively steady (Steinfeld et al., 2010). This progress has been in no small part due to improvements to genetic potential across many traits. As industries around the world work to produce high-quality protein while balancing economics, animal well-being, and environmental impact, genetic improvement will be an even more important driver. Genetic improvements are complementary and often additive to environmental and management interventions. Genetic progress is cumulative and permanent, meaning that improvements are not lost between generations and add up over time. This makes breeding a long-term solution to addressing ­problems or driving further improvement, provided the traits are heritable. Genetic progress often happens more slowly than management, medical, or nutritional interventions, but provides a long-term approach to improving populations, while other approaches bridge in the short term.

Breeding programs evolve over time and can include novel traits of interest in improvement plans as economic or social signals exist to do so, or tools evolve to enable measurement and testing. While early genetic evaluations focused primarily on growth and productivity traits, contemporary iterations include economically important traits from across an animal’s lifetime and across cow-calf, backgrounding, feeding, and meat packing sectors. New measurement systems and economic drivers will enable and incentivize the collection of sustainability-related phenotypes like enteric methane emission and grazing feed intake. The selection tools developed by these outcomes will be essential for inclusion in selection indices that define larger population breeding goals.

The tools at breeders’ disposal have enabled increased selection accuracy (Henderson, 1975; Meuwissen et al., 2001; Legarra et al., 2014), decreased generation interval, and ultimately accelerated genetic gain. The suite of tools used for genomic selection is largely a mature technology. As such, future innovations will rely on expanding the suite of traits with genetic predictions rather than to the genetic prediction machinery itself. Genetic evaluations rely on large numbers of phenotypes to be measured on genotyped or pedigreed animals to produce selection tools. Innovations in technology for observing and measuring new phenotypes from across the beef value chain will be instrumental. In addition to an expanded genetic toolbox, advanced reproductive technologies and breeding strategies are accelerating genetic progress. These tools like artificial insemination (AI), in vitro embryo production (IVP), and embryo transfer (ET) allow for the rapid propagation of elite genetics. These technologies are also being deployed across emerging systems like Beef-on-Dairy crossbreeding where production efficiency and scale have multiplicative effects on sustainability outcomes. Finally, the Global South’s rapidly evolving economy has driven demand for high-quality protein and consequently to improvements to their beef production system. The expansion and intensification of these beef ­production systems will have enormous impacts on global sustainability outcomes. The role that genetics will play in improving the sustainability of beef production worldwide will look different across systems. The genetic improvement strategies deployed in the Global North will look different from the Global South. Regardless of region or production system, breeding interventions should align with the Food and Agriculture Organization’s (FAO) tenets of sustainability (FAO, 2021). This means that breeding programs should be (1) environmentally non-degrading, (2) economically viable, (3) conserve genetic resources, (4) be technically appropriate, and (5) socially acceptable (Figure 1).

**Figure 1.**
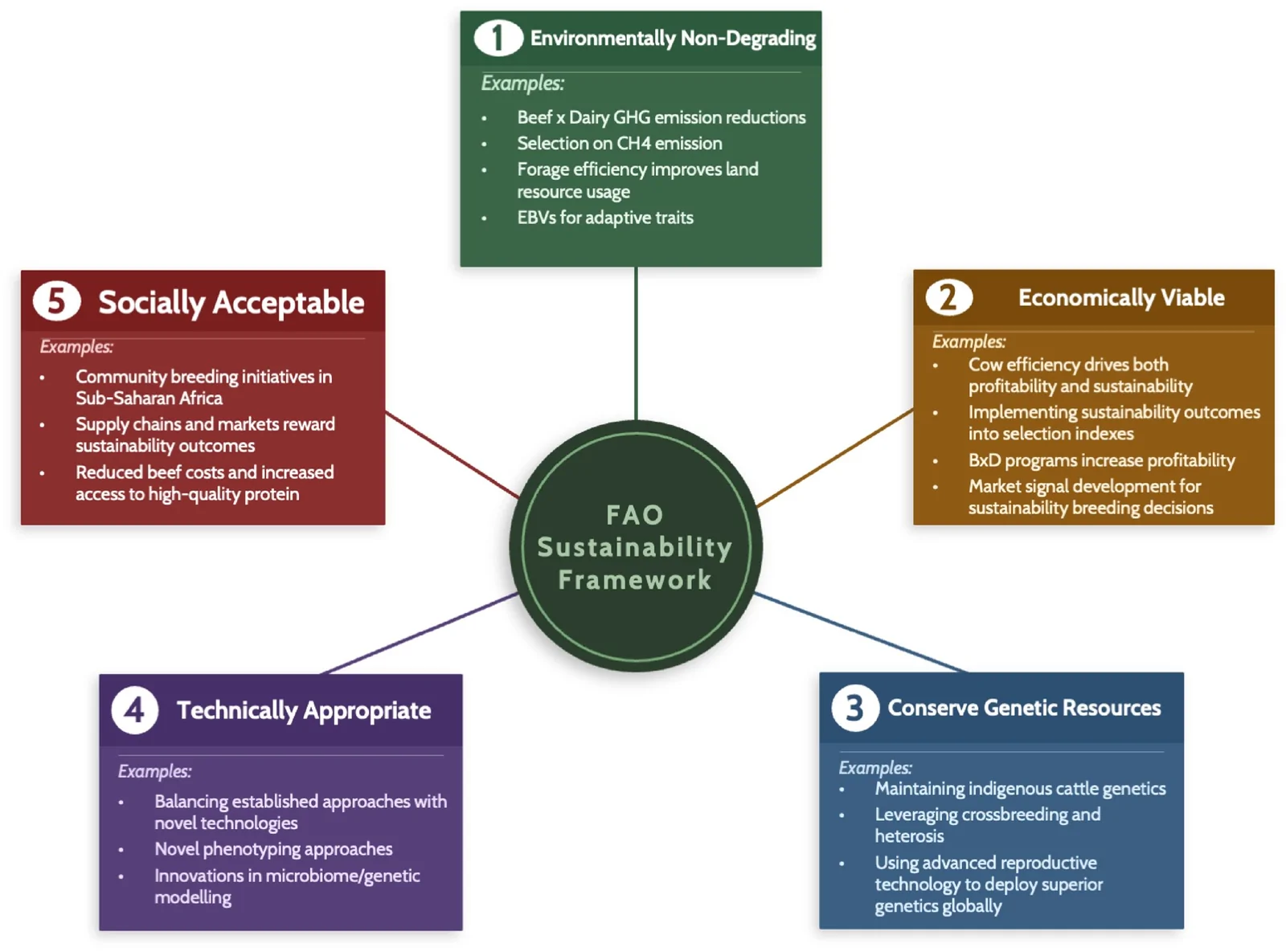
Diagram featuring the UN Food and Agriculture Organization’s definition of sustainable interventions with applied examples from this review (FAO, 2021).

The role that genetics plays in driving sustainability improvements to the beef industry is highly variable depending on the specific goals and areas of interest. Most of the beef industry’s greenhouse gas (GHG) footprint stems from enteric methane. Multiple pathways exist for genetics to drive reductions in emissions, via either productivity gains that improve animal efficiency, or by directly selecting on methane.

This paper serves as an overview of how genetic improvement programs can drive improvements in beef cattle sustainability. We review current activities across a diverse array of production systems as well as emerging solutions for continued progress.

## Genetic Improvement and Beef Sustainability

### Sustainability through productivity improvements

The goals of efficient and sustainable genetic improvement programs should include improvements to productivity with little or no negative impact on the environment, improve fitness-related traits such as reproductive ability, disease resistance, robustness, welfare traits, and should be economically profitable and socially acceptable.

Across beef production systems worldwide, most life cycle analyses assign over 80% of the industry’s GHG footprint to the grazing cow-calf sector (Rotz, 2023). Grazing cows are the foundation of the global beef industry, but are also its largest emitters, as enteric emissions are significantly higher for animals consuming forage compared to those fed concentrate in feedlot systems (Johnson and Johnson, 1995). As such, genetic improvements that impact the efficiency of grazing cows would have an outsized impact on the environmental footprint of beef production. Gains in forage-based efficiency would be even more impactful in grass finishing systems like New Zealand, the United Kingdom, and Ireland. To date, the U.S. Roundtable for Sustainable Beef and other sister organizations around the world have concentrated sustainability improvements in the cow-calf sector, primarily expanding the adoption of adaptive grazing management plans (Tang et al., 2024). Grazing management is only half of the equation in optimizing the efficiency of forage utilization. It relies equally on breeding efficient cows that are well-suited to perform in their environmental context. Efficiency is much more than feed conversion. Traits like fertility, health, and longevity all impact the efficiency of beef systems whose core function is to make the most effective long-term use of grazing lands and harvested feed. Cows that fail to generate sufficient output for their intake, open cows, or cows that fall out of production prematurely diminish the sustainability of a ­production system. Cow longevity is central to cow-calf sustainability and profitability, as long-lived females can spread their development costs and GHG footprint across more calves. Regardless of the cause, cows that fall out of the herd prematurely fail to return on their up-front investment. All of the traits that contribute to longevity—from fertility to structural soundness to disposition—have distinct values to the economic and environmental sustainability outcomes. The diverse ways that cow efficiency can be defined are represented in Figure 2 and detailed in (Rowan, 2022) .

**Figure 2.**
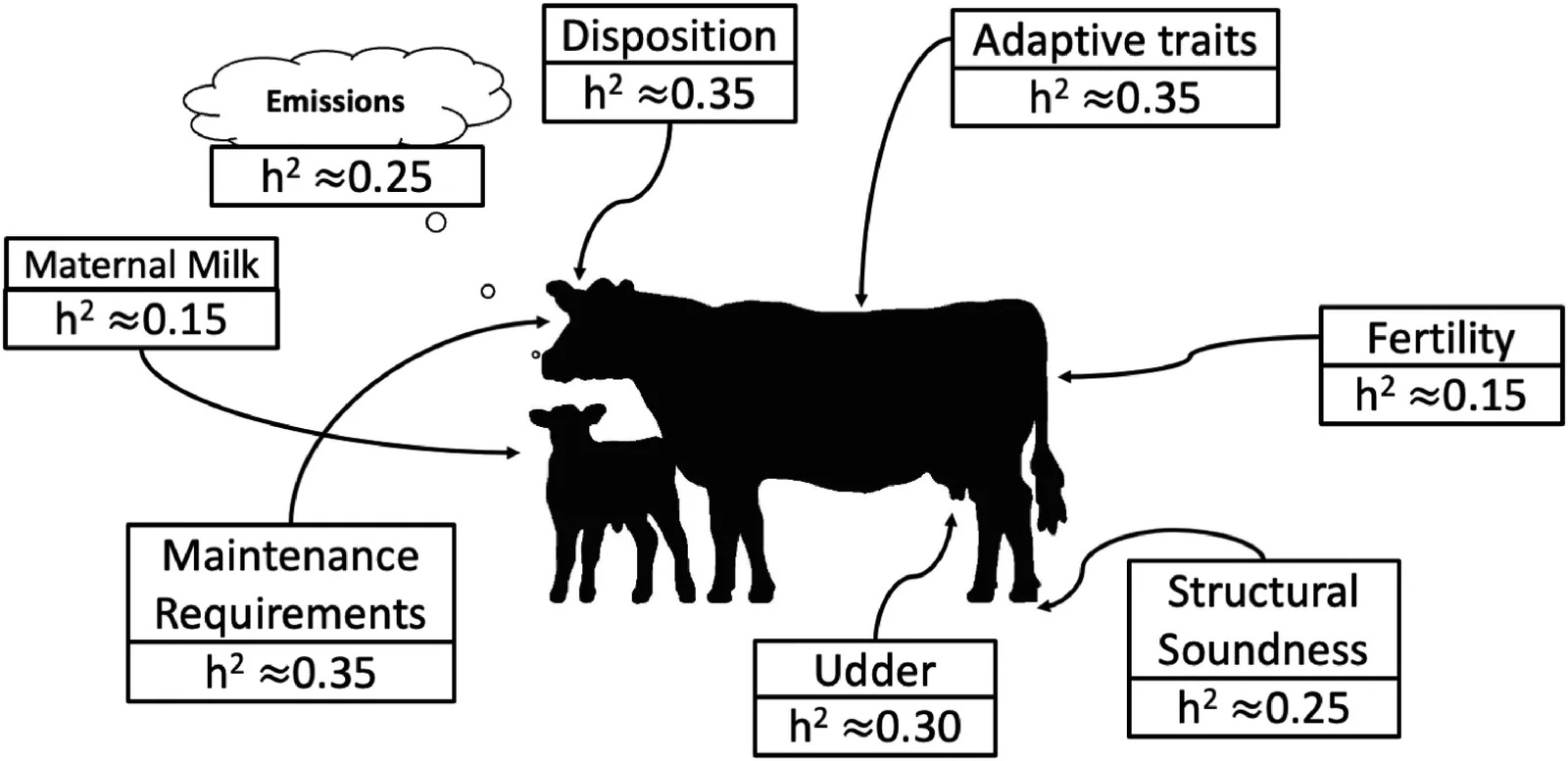
The many drivers of cow efficiency and their approximate heritabilities. Heritabilities for traits with EPDs reported by American Angus are reported here. Heritabilities for emissions and maintenance requirements are reported from Dressler et al. (2024). Figure is adapted from Rowan (2022).

Selection for increased growth and carcass weights has led to a general increase in cow size. Genetic correlations between mature cow weight and growth traits are high (0.67 for weaning weight and 0.74 for yearling weight in American Angus) (Ojo et al., 2025). Generally, heavier mature weights are associated with greater maintenance energy requirements. Consequently, system-wide efficiency tends to be higher when more, smaller cows are stocked on the same land footprint (Bir et al., 2018). Beyond cow size, lactation potential is also a critical element of forage-based efficiency (Montaño-Bermudez et al., 1990).

Genetic selection tools for mature weight and maternal weaning weight (i.e., milk) are published by many major genetic evaluations, allowing producers to match energy needs to resource availability. Other genetic evaluations have also expanded to include traits related to adaptability that help match genetics to environmental stressors. This includes genomic predictions for early-season hair shedding (Durbin et al., 2020), an indicator of adaptation to heat and toxic fescue stress, for pulmonary arterial pressure (PAP) (Pauling et al., 2018), an indicator of adaptation to high elevation, and for high immune response (HIR), a selection target for improving health outcomes.

Tools for predicting feed efficiency genetic potential exist across major genetic evaluations but are focused on feedlot and finishing settings. These are calculated based on phenotypes from structured feed intake trials of growing animals. While these selection tools are enormously useful, the phenotypes likely fail to capture the full spectrum of cow efficiency related to grazing behavior, environmental adaptation, or forage selectivity (Rowan, 2022). Further, emerging evidence suggests that cow mature size is not a perfect indicator of forage intake. Consequently, the development of more robust tools that directly measure animal metabolism and grazing behaviors could more accurately represent likely forage intake.

### Direct selection on enteric methane emissions

Selection programs around the world have increased interest in adding estimated breeding values (EBVs) for methane emission to their existing suite of traits. Methane traits are moderately heritable, with estimates ranging from 0.15 to 0.45 across various studies of beef cattle (Dressler et al., 2024). Direct methane emission has a limited utility in most operations’ breeding objectives as a target trait, though some external actors, like governments, may be interested in reducing gross CH_4_ emissions. Methane emission represents wasted energy in the enteric fermentation process, meaning that direct selection may improve animals’ ability to effectively mobilize feed energy. Despite limited signals for producers to select directly on methane emission, the emergence of market signals in the future could alter these priorities and incentivize a place in future breeding objectives.

Other actors, like supply chains focus on CH_4_ intensity, which are emissions scaled by animal output (e.g., live weight or carcass weight), as these are the units that they report emissions on (Ryan et al., 2022). This means that even in the absence of tools for direct selection on CH_4_, producers can continue to reduce GHG footprints via sustained productivity improvements in output traits. Methane yield measurements scale gross emissions by dry matter intake, two traits that are highly correlated. Finally, deviations from expected emissions given body weight and feed intake can be represented as residual methane, like residual feed intake (RFI) calculations for feed efficiency (Figure 3). While residual CH_4_ may help identify phenotypic outliers of emissions of a given body size, it is unlikely to be directly selected on when other measures of emission, intake, and gain can be directly modeled in an economic selection index.

**Figure 3.**
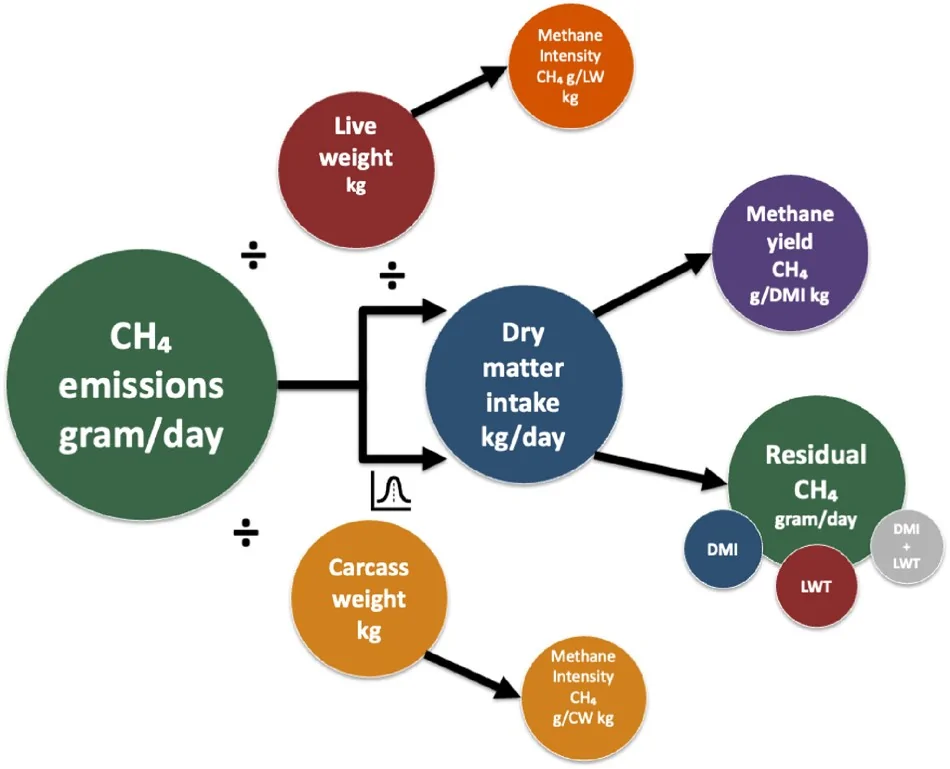
Depictions of the different possible enteric methane-related phenotypes of interest in beef cattle. In each case, derived traits consist of CH_4_ emissions scaled by either outputs (CH_4_ intensity), feed intake (CH_4_ yield), or derived as a statistical residual value. Adapted from Ryan et al. (2022).

While governments and supply chains may be more interested in singular outcomes of genetic progress on methane emissions, beef operations balance a range of traits in their selection decisions, from fertility to health to productivity. Ideally, they would do so using economic selection indices that weight trait EBVs by their relative economic importance to a larger breeding objective (Enns and Nicoll, 2008). Depending on the marketing endpoint (i.e., carcass or live calf) and decision to retain replacement heifers, indices allow producers to make genetic progress on the full suite of traits in a way that is optimized to maximize profit. The usefulness of CH_4_ emission genomic predictions will ultimately be when used in selection indices. Selection also allows for breeding programs to account for correlated responses that may be unfavorable between traits. Long-term selection experiments in sheep suggest that there are relatively few antagonistic genetic correlations between CH_4_ and other economically-relevant traits (Hickey et al., 2022), but indices can handle these multiple drivers and arrive at an economic optimum. For these solutions to be integrated into breeding programs and their corresponding selection indices, it will require that supply chains present adequate market signals to producers to integrate sustainability into their breeding programs.

Reducing emissions from cattle populations will differ regionally. Countries in the Global South will likely lean on sustainable intensification and enhanced productivity to reduce emissions intensities. In countries where enteric emissions make up large percentages of the national GHG inventory (e.g., Ireland, New Zealand, the United Kingdom), direct selection on CH_4_ traits may be more attractive. Systems that primarily utilize grass finishing rather than concentrate could further reduce emissions footprints via improvements to growth rates, and consequently reducing days on feed. This is particularly important in the tropics where beef production is rapidly expanding and improving. Across all systems, continued genetic progress on efficiency traits, especially those related to grazing cows, will be the most important driver of reducing emissions.

Developing genetic selection tools for emissions relies on generating tens of thousands of phenotypic records and genotypes across pedigreed populations. Recent investments by the Global Methane Genetics Initiative (GMG) aim to phenotype over 100,000 animals internationally for use as a reference training population. This will include beef cattle in Oceania, North America, South America, Europe, and Africa and allow for comparisons across cattle subspecies, breeds, and production systems (de Hass et al., 2025).

### Genetic improvement programs in Sub-Saharan Africa’s beef industry

Whereas cow nutrient requirements and weight outputs are the focus of productivity-related sustainability improvements across the Global North, in Sub-Saharan Africa, maintaining the unique genetic characteristics of the indigenous beef cattle breeds is a central challenge. Indigenous cattle have evolved remarkable adaptations to the region’s harsh and variable environments, including disease and parasite tolerance and adaptation to low-quality forage and heat stress. This makes the preservation and enhancement of these adaptive traits essential in conjunction with productivity gains, the combined objective of genetic improvement programs in the region. Breeding ­strategies that build on this foundation of resilience, using community-based programs to select within indigenous populations for improved productivity while safeguarding disease tolerance and environmental fit, represent the most sustainable path forward for a sector defined by climate variability, limited veterinary infrastructure, and smallholder production systems. Sacrificing this adaptation and genetic diversity could have profound negative consequences on the sustainability of the system. Although breed improvement in the beef sector in Africa is limited, a few examples demonstrate both productivity gains and sustainability benefits of such programs. These programs illustrate the foundational importance of matching genetics to environment, as well as the importance of community-led breeding programs. The impacts of community breeding programs and other developments in cattle breeding are reviewed at length in Ibeagha-Awemu et al. (2026).

Despite the demonstrated potential of community-based breeding programs in Sub-Saharan Africa, systemic constraints continue to limit their scale and impact across the region. Breeding approaches vary widely between commercial and smallholder systems and tend to be largely unstructured (Rewe et al., 2009). Institutional gaps inhibit widescale data recording that would enable development of genetic improvement programs. Accelerating performance recording infrastructure would provide opportunities for recording traits that have both productivity and sustainability impacts. In many countries, historical investment has disproportionately favored crossbreeding for dairy improvement, leaving beef genetic improvement comparatively underdeveloped despite its substantially larger land and environmental footprint (Ouédraogo et al., 2021).

Productivity gains in African cattle breeds will also require addressing significant gaps in data infrastructure and policy. African cattle remain underrepresented in global genomic reference datasets, limiting the precision and applicability of genomic selection tools across the continent (Ibeagha-Awemu et al., 2026). Integration of genomic technologies is further constrained by limited infrastructure, insufficient technical capacity, and weak performance recording systems. At the policy level, frameworks governing data sharing, intellectual property, and conservation of indigenous genetic resources are often underdeveloped, resulting in the gradual erosion of the adaptive diversity that makes these breeds uniquely suited to their production environments. Before sustainability traits can be directly improved in Sub-Saharan Africa, investment in phenotypic data collection, genomic infrastructure, and supportive policy are essential.

### Beef × dairy and the contribution of selection to reducing environmental impact

Beef × dairy production provides an interesting case study for the potential to increase beef sustainability through genetic improvement. This is made possible by the higher rates of AI and routine data collection found in many dairy systems. Dairy herds are a significant source of beef but were not designed to optimize the productivity of the beef supply chain. The introduction of sexed semen in the early 2000s, combined with the widespread implementation of genomics by dairy cattle breeders, drove rapid genetic progress for dairy-focused traits while only producing enough females needed as replacements in the milking herd. This led to a shift in the mating strategy for dairies around the world, where a growing proportion of females in the herd were bred to beef genetics, resulting in a beef × dairy (B×D) calf. This ­crossbred animal is typically more efficient and more productive in beef growing and finishing systems and thus better suited to the beef supply chain (Figure 4). The crossbred calf’s improved growth efficiency also reduces its carbon footprint—spending less days on feed, resulting in a decreased emission intensity.

**Figure 4.**
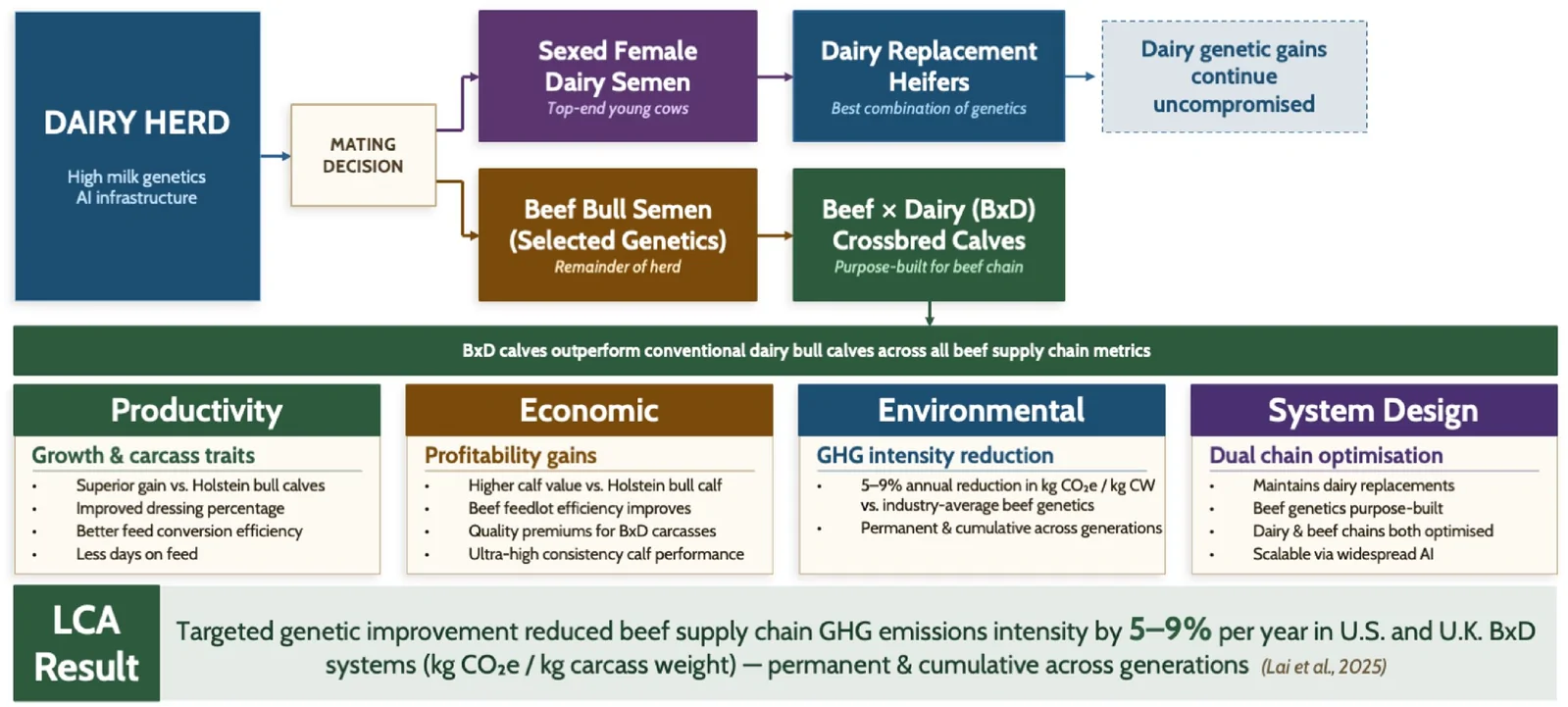
Diagram of beef-on-dairy system implementation and the general benefits of beef sired calves in U.S. and U.K. supply chains. Based on the findings of the beef-on-dairy lifecycle analysis described in Lai and Cleveland (2025).

The choice of mating strategy can profoundly affect the magnitude of the impact that genetic improvement can deliver. The shift to the use of both sexed female dairy and beef genetics by farmers was a strategic decision to accelerate dairy genetic improvement, but in doing so, created a demand for improved beef genetics, spurring the development of breeding programs targeting B×D production. Beef genetic improvement implemented in such a designed mating system has the potential to improve traits important to the beef supply chain (e.g., sire conception rate, growth, efficiency, and carcass characteristics), without compromising genetic gains for traits important to the dairy supply chain. Dairy and beef production are both optimized. Calves that were once seen as by-products of dairy production are becoming highly desirable as beef animals due to the focus on B×D genetic improvement.

Sexed and beef mating strategies, and designed breeding programs for B×D genetics, provide a path to more sustainable beef production for dairy farmers. This approach has increased productivity and made farmers more profitable (Basiel and Felix, 2022). There has also been the added benefit of reducing environmental impact driven by resulting productivity gains. A full Lifecycle Assessment of U.S. and U.K. B×D systems showed that targeted genetic improvement had the potential to reduce beef supply chain GHG emissions intensity (kg CO_2_ equivalents/kg carcass weight) by 5% to 9% per year (Lai et al., 2025; Lai and Cleveland, 2025), compared to industry average genetics. Changes from genetic improvement are incremental per generation, but permanent and cumulative over multiple generations, making genetic improvement an important sustainability solution. Given the popularity of B×D production systems around the world, continued selection for more productive and profitable B×D genetics is integral to developing more sustainable beef value chains.

## Current and Emerging Solutions

### Deploying genetics: importance of advanced reproductive technologies

The real power of genetic improvement comes from the cumulative and permanent nature of its effect. These effects can transform animal populations over time through selective breeding programs. Reproductive technologies are essential tools for disseminating improved genetics at the scale required to have a meaningful impact. However, progress to implement new technologies in extensive beef systems has been relatively slow compared to other livestock species, like dairy cattle, pigs, or poultry, due to logistical implementation challenges, inefficiencies, or resistance to change.

Reproductive technologies enable the dissemination of improved genetics and ensure that beef producers are leveraging their existing resources to their fullest potential (Lamb et al., 2016). While not universally applied to beef production, AI, estrus synchronization, and, more recently, timed AI (TAI) have all been used to disseminate genetic improvement from breeding programs to commercial beef producers. Estrus synchronization and TAI made it possible to implement AI in more systems, which led to faster rates of genetic gain than were possible using traditional approaches, from greater access to better genetics. Development of technology to efficiently create and cryopreserve embryos (e.g., multiple ovulation and ET and in vitro fertilization) provided a way to accelerate genetic progress by multiplying and disseminating the best genetics at rates not possible using traditional AI, though implementation in commercial operations remains limited. Sex-selected semen has been used in both AI and embryo approaches to combine improved genetics with optimized dissemination to further enhance the productivity and sustainability of the system under selection (e.g., beef × dairy).

New and emerging technologies may also play a role in the more rapid dissemination of improved traits, but the cost of implementation needs to be balanced against the value of the outcome. Somatic cell nuclear transfer, or cloning, has been used in numerous species, including beef cattle, and can increase selection intensity, thereby accelerating genetic gain through duplication of elite male and female genetics. In practice, the cloned animals need to be of sufficient genetic level to overcome the time lag inherent in the cloning process and, thus, cloning has largely not been impactful to the beef industry. Genetic modification or gene editing (GE) has emerged as an important approach to address the improvement of traits that are more difficult to target through traditional selection programs. To date, GE has been used in multiple species, including beef cattle, and appears to be mostly targeted to disease and welfare traits in major livestock species (Van Eenennaam, 2025), which would be particularly useful in improving productivity and reducing environmental impact (Thoma et al., 2025).

### Novel phenotyping and evaluation methods

Genetic evaluations rely on the routine collection of phenotypes to function. Novel phenotype capture will be the driving forces behind improvements in future genetic selection programs that focus on improving sustainability across the industry.

Supply chains and governments have put pressure on beef production systems to reduce their GHG footprint, driving interest in direct genetic selection for permanent and cumulative reductions in CH_4_ emissions. Historically, measuring individual animals for enteric CH_4_ emission has been expensive, low throughput, and out of environmental context. Developments in gas flux (i.e., GreenFeed emissions monitoring system) and concentration (i.e., sniffers) measurement devices have enabled more widespread phenotyping, particularly in grazing settings. These phenotyping platforms also offer opportunities to capture indirect measures of cow efficiency like carbon dioxide emissions and metabolic heat production (Dressler et al., 2024).

Enteric emissions and efficiency phenotypes are unique in their expression as they are animal traits that are largely driven by the commensal microbes that colonize the rumen. There are known relationships between animal genetics and the existence and abundance of particular microbial communities responsible for feed efficiency and methane emissions (Martínez-Álvaro et al., 2022). These relationships also offer opportunities to jointly model host genetic potential and microbiome composition for accurate phenotypic predictions that could be used to develop more individualized animal management approaches. (Alemu et al., 2025). Sampling rumen contents can be invasive and likely does not fit into the routine production practices of most beef operations around the world. Emerging evidence suggests that oral swabs of the mouths of ruminants may be a worthwhile substitute for orogastric tubing. Even though the communities identified in each sampling medium are different, both sample types account for similar levels of variation in feed efficiency and CH_4_ emissions when simultaneously modeled alongside host genetics.

## Conclusion

Genetic improvement represents one of the most powerful, permanent, and scalable tools available to reduce the environmental footprint of global beef production. Unlike management or nutritional interventions that must be reapplied each generation, genetic gains are cumulative and self-sustaining, compounding in value across time and populations. As reviewed here, multiple pathways exist for genetics to drive sustainability improvements: efficiency gains in grazing cow-calf systems, direct selection on enteric methane emission, indigenous breed conservation in Sub-Saharan Africa, and the rapid adoption of beef × dairy crossbreeding in dairy-integrated systems. In all cases, the value of genetic improvement is multiplied by robust reproductive technologies that disseminate superior genetics broadly across commercial populations. Looking forward, the field faces important challenges and opportunities. Novel phenotyping approaches, new genomic prediction methodologies, and advanced reproductive technologies will help better identify and deploy these genetics across global beef systems. Ultimately, realizing the full sustainability potential of genetic improvement will require alignment of economic incentives, regulatory frameworks, and breeding objectives across the diverse stakeholders of the global beef value chain.

## Author Biographies

**Dr. Matthew A. Cleveland**, a geneticist by training, is passionate about using technology and innovation to create a more sustainable food supply. He currently leads Sustainability at ABS Global, a Genus plc company, a world leader in animal genetic improvement. Matthew is responsible for ABS’ overall beef and dairy sustainability strategy, including sustainability product development and positioning, for the global bovine business, to ensure ABS can continue to deliver more profitable and sustainable beef and dairy genetics to customers around the world. Matthew speaks extensively to technical and non-technical international audiences about the role of livestock production in sustainably feeding the growing demand for animal source proteins. He currently sits on the Executive Committee of the Global Roundtable for Sustainable Beef and previously served on the board of the U.S. Roundtable for Sustainable Beef. He has authored numerous papers related to innovation, genetic improvement, and sustainability in livestock. Having previously led the global beef genetic program at ABS from 2014 to 2024, he also held roles in genetic and computational R&D. Matthew has a PhD from Colorado State University, and an MBA from the University of Wisconsin, Madison.

**Dr. Julie Ojango** is a Senior Scientist at the International Livestock Research Institute (ILRI) with more than 20 years in livestock genetics. She leads interdisciplinary research combining biological, socio-economic, and environmental perspectives to strengthen sustainable cattle, sheep, and goat breeding in low and middle-income countries through climate-smart innovations, livestock data systems, and evidence-based policy for smallholder and pastoralist communities. She also trains agricultural professionals across Africa and Southeast Asia in livestock genetic resources. An adjunct professor at the University of Tennessee, she serves on ILRI’s Animal Care and Use Committee and the International Dairy Federation’s steering committee, and previously lectured at Egerton University, Kenya. She holds an MSc from the University of Nairobi and a PhD in Animal Breeding and Genetics from the University of London.

**Dr. Raphael Mrode** holds a joint position as Professor of Quantitative Genetics and Genomics at the Scotland Rural College (SRUC), United Kingdom, and Principal Scientist in Quantitative Dairy Cattle genetics at the International Livestock Research Institute (ILRI), Kenya. He has been involved in the development and research underpinning genetic and genomic evaluations in dairy and beef cattle, goats and sheep in the United Kingdom over 30 years and in the development of innovative genomic approaches to livestock genetic improvement in low-input environments at ILRI. He has been a member of technical committee of INTERBULL, the organization in charge of international dairy cattle evaluations since 2004, an auditor for the International Committee for Animal Recording (ICAR) and research consultant supporting the FAO/IAEA coordinated research project on the Application of Nuclear and Genomic Tools. He is the author of the text, “Linear Models for the Prediction of Breeding Values,” now in its fourth edition, co-author of two other textbooks, author of two book chapters, have over 100 peer-reviewed papers and more 300 conference papers. He has been a member of the Editorial Board, *American Journal of Animal Science* and he is the Assistant Section Editor on Animal genetics for the journal, *Animal*.

**Mike Coffey** is a Professor in Livestock informatics at Scotland’s Rural College (SRUC) and Head of Edinburgh Genetic Evaluation Services (EGENES). His main areas of research interest lie in the genetic and genomic improvement of farmed livestock for traits valuable to the animal, the farmer, and society. This started in dairy cattle but has expanded to include beef, sheep, goats, and pigs. Recent socio-economic developments and their resulting price spikes have sharply focused attention on efficiency traits and environmental impact. Recently, his group have begun exploring the use of Deep Learning to predict new phenotypes from milk mid infra-red (MIR) spectral data. Successes so far include predicting pregnancy status and bTB status and extracting new phenotypes automatically from sheep CT scans and goat and cow udder images. They are expanding traits of interest to include Johnes disease, BVD and contaminants in milk. They are also looking to predict feed intake and methane emissions using MIR data in an international consortium.

**Dr. Troy Rowan** is an Assistant Professor in the Department of Animal Science at the University of Tennessee, where his research program focuses on the application of genomic and quantitative genetic tools to improve the efficiency, sustainability, and profitability of beef cattle production systems. His work uses large-scale genomic datasets and novel phenotypes to identify the genetics underlying grazing cow efficiency, adaptation, and resilience. His group’s research spans the continuum from fundamental biological discovery to applied genomic selection strategies, all with the goal of developing actionable tools for seedstock producers, breed associations, and commercial cow-calf operations. In addition to his research, Dr. Rowan is engaged in extension and outreach efforts aimed at communicating the value of genomic technologies to beef producers around the globe. Troy’s extension work supports the Tennessee Master Beef Producer Program and other national organizations (the Beef Improvement Federation, NCBA, and U.S. Roundtable for Sustainable Beef).
